# Upregulation of Gelatinases and Epithelial–Mesenchymal Transition in Small Airway Remodeling Associated with Chronic Exposure to Wood Smoke

**DOI:** 10.1371/journal.pone.0096708

**Published:** 2014-05-06

**Authors:** Yimin Zou, Shaoxing Li, Weifeng Zou, Guoping Hu, Yumin Zhou, Gongyong Peng, Fang He, Bing Li, Pixin Ran

**Affiliations:** 1 The State Key Laboratory of Respiratory Disease, Guangzhou Institute of Respiratory Diseases, the First Affiliated Hospital, Guangzhou Medical University, Guangzhou, Guangdong, China; 2 Guangzhou Chest Hospital, Guangzhou Medical University, Guangzhou, Guangdong, China; 3 The Third Affiliated Hospital, Guangzhou Medical University, Guangzhou, Guangdong, China; 4 The Research Center of Experiment Medicine, Guangzhou Medical University, Guangzhou, Guangdong, China; Helmholtz Zentrum München/Ludwig-Maximilians-University Munich, Germany

## Abstract

**Background:**

Peribronchiolar fibrosis is an important feature of small airway remodeling (SAR) in cigarette smoke-induced COPD. The aim of this study was to investigate the role of gelatinases (MMP9, MMP2) and epithelial-mesenchymal transition (EMT) in SAR related to wood smoke (WS) exposure in a rat model.

**Methods:**

Forty-eight female Sprague-Dawley rats were randomly divided into the WS group, the cigarette smoke (CS) group and the clean air control group. After 4 to 7 months of smoke exposure, lung tissues were examined with morphometric measurements, immunohistochemistry and Western blotting. Serum MMP9 and TIMP1 concentrations were detected by ELISA. In vitro, primary rat tracheal epithelial cells were stimulated with wood smoke condensate for 7 days.

**Results:**

The COPD-like pathological alterations in rats exposed chronically to WS were similar to those exposed to CS; the area of collagen deposition was significantly increased in the small airway walls of those exposed to WS or CS for 7 months. The expression of gelatinases in rats induced by WS or CS exposure was markedly increased in whole lung tissue, and immunohistochemistry showed that MMP9, MMP2 and TIMP1 were primarily expressed in the airway epithelium. The serum levels of MMP9 and TIMP1 were significantly higher in rats secondary to WS or CS exposure. Few cells that double immunostained for E-cadherin and vimentin were observed in the airway subepithelium of rats exposed to WS for 7 months (only 3 of these 8 rats). In vitro, the expression of MMP9 and MMP2 proteins was upregulated in primary rat tracheal epithelial cells following exposure to wood smoke condensate for 7 days by Western blotting; positive immunofluorescent staining for vimentin and type I collagen was also observed.

**Conclusions:**

These findings suggest that the upregulation of gelatinases and EMT might play a role in SAR in COPD associated with chronic exposure to wood smoke.

## Introduction

Chronic obstructive pulmonary disease (COPD) is characterized by persistent airflow limitation, which is usually progressive and classically involves clinical or pathological presentations (chronic bronchitis, emphysema and small airway disease) [1]. Cigarette smoking accounts for approximately 80–90% of COPD cases in the United States [2]. Biomass smoke (wood, dung, or straw) derived from household cooking and heating is another leading cause of COPD, mostly in developing countries [3]. Patients exposed to biomass smoke can present with emphysema and other lesions observed in cigarette smokers, but they show more lung fibrosis than cigarette smokers [4]; these indications are unclear in the pathogenesis of COPD secondary to biomass smoke exposure.

Small airway remodeling (SAR), rather than emphysema, is the major cause of airflow limitation in COPD [5]. Peribronchiolar fibrosis has been speculated as the key factor in the fixed airway obstruction in the small airways of patients with COPD associated with cigarette smoke [6]. Tissue fibrosis occurs when equilibrium is lost between the deposition and degradation of the extracellular matrix (ECM). Matrix metalloproteinases are a family of zinc-dependent endopeptidases that are involved in the breakdown and remodeling of the ECM [7].

Very little is known about the role of matrix metalloproteinases in the SAR in COPD, although there is a widespread belief that matrix metalloproteinases account for matrix disruption and airspace enlargement in the pathogenesis of emphysema [8]. Some studies have shown the increased expression and activity of MMP9 and MMP2 in the sputum, bronchoalveolar lavage fluid and peripheral blood from patients with COPD, and MMP9 and MMP2 may contribute to the severity of airflow limitation during COPD progression [9], [10]. TIMP1 is a natural inhibitor of MMPs (mainly MMP9), which inhibits matrix metalloproteinases in a 1: 1 stoichiometric and reversible manner [11]. An imbalance of MMP9 with TIMP1 might be involved in the pathogenesis of airway obstruction in COPD [12]. However, the induced sputum is derived from the central airways with little or no contribution from the peripheral airways; and bronchoalveolar lavage fluid cannot be discriminated by its source, which includes the individual components of the distal airway and alveolar compartments [13]. The role of MMP9 and MMP2 in SAR in COPD needs to be studied through direct analyses of airway tissues.

Fibroblasts are believed to be the major cells responsible for the production and maintenance of the ECM [14]. Recently, one potential mechanism contributing to airway fibrosis is the transition of airway epithelial cells to fibroblast phenotypes, a process termed epithelial-mesenchymal transition (EMT) [15]. Evidence for EMT in the airway remodeling in COPD patients secondary to cigarette smoke has been reported by Sohal [16] and Milara [17]. However, whether EMT is present in the SAR in COPD induced by biomass smoke exposure remains to be elucidated.

There are few animal models of COPD related to biomass smoke exposure. One recent study has shown the role of MMP9 and MMP2 in emphysema secondary to wood smoke (WS) exposure in a guinea pig model [18]. The present work was to study the role of gelatinases and EMT in SAR in COPD associated with chronic exposure to WS in a rat model.

## Materials and Methods

### Ethics Statement

All animal experiments were approved by the Local Ethics Committee of Guangzhou Medical University.

### Experimental Animals

Forty-eight female Sprague-Dawley rats (body weight 180–220 g, 8–9 weeks old) were housed in the laboratory animal center of Guangzhou Medical University. Rats were randomly divided into the WS group, the cigarette smoke (CS) group and the clean air control group.

### Exposure to WS

**Figure 1:**
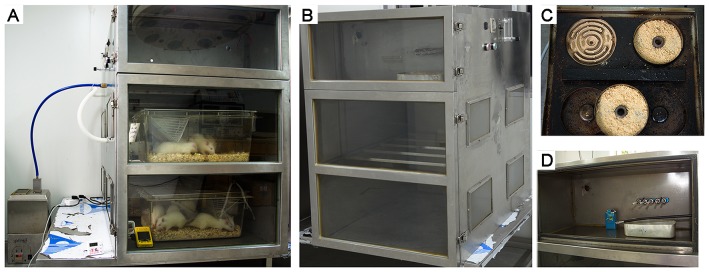
The whole-body smoke exposure apparatus. (A) The WS exposure apparatus primarily consisted of a wood burning stove, a piston pump and an inhalation chamber. (B) The CS exposure apparatus primarily consisted of a cigarette burning box, a piston pump and an inhalation chamber. (C) The wood burning stove had four independent heating wires (300 W), and the small iron trays were placed on the heating wire to smolder the wood sawdust. (D) The cigarette burning box had five cigarette holders.

Rats were exposed to smoke produced by smoldering China fir sawdust (10 g/per time) for 45 minutes, 4 times per day, 7 days per week for 4 to 7 months. The whole-body WS exposure apparatus primarily consisted of a wood burning stove, a piston pump and an inhalation chamber (Figures 1A and 1C). The WS generated by the burning stove was sent into the inhalation chamber through a piston pump (2.5 L/min). The fresh air was sent into the inhalation chamber by a mini air pump (1.5 L/min). The size of the inhalation chamber was 80×60×60 cm. In this manner, WS flowed from the burning stove to the inhalation chamber and then to the outside.

The CO and O_2_ concentrations in the inhalation chamber were monitored with a detector (M40 Multi-Gas Monitor; Oakdale, PA, USA); PM10 concentrations were measured with an automatic dust monitor (P-5L2C; Peking, China). During WS exposure, the concentrations of CO, O_2_ and PM10 were 539.3±309.8 ppm, 20.2±0.4% and 27.6±12.1 mg/m^3^, respectively.

### Exposure to CS

Rats were exposed to the smoke generated by 5 cigarettes (Cocopalm, Guangzhou, China; tar 12 mg and nicotine 1.2 mg) for 45 minutes, 4 times per day, 7 days per week for 4 to 7 months. The whole-body CS exposure apparatus primarily consisted of a cigarette burning box, a piston pump and an inhalation chamber (Figures 1B and 1D). The mainstream smoke was drawn into the inhalation chamber by a piston pump, according to the Federal Trade Commission protocol (1 puff/min of 2 sec duration and 35 ml volume); the sidestream smoke was sent into the inhalation chamber by a fan. A mini air pump was used for the air supplement into the inhalation chamber (1.5 L/min). During CS exposure, the concentrations of CO, O_2_ and PM10 were 467.2±98.1 ppm, 20.1±0.4% and 20.5±9.9 mg/m^3^, respectively.

### Sampling the Lung Tissues and Blood

Rats were sacrificed by the intraperitoneal injection of sodium pentobarbital (50 mg/kg). The left lungs were inflated and fixed using 4% phosphate-buffered formaldehyde (pH 7.40) at 25 cm H_2_O pressure for 24 h. The lungs were then embedded in paraffin and cut into 4 µm thick sections. The right lung tissues were snap frozen in liquid nitrogen and stored at −80°C for Western blot analysis. Blood samples were taken from the inferior vena cava, and the serum was stored at −80°C for ELISA.

### Morphometric Measurements

To avoid observer bias, all microscope slides were coded before analysis by one observer and were read blindly. Photographs were taken on a Zeiss Axio Imager 2 Microscope (Carl Zeiss, Germany), and morphometric analyses were quantified using Image-Pro Plus (IPP) 6.0 software (Media Cybernetics, Silver Spring, USA).

Airspace size was quantified in lung tissues stained with H&E using the mean linear intercept [19]. The thickness of the small airway wall was analyzed according to methods described previously [20]. Small airways cut transversely and with a basement membrane perimeter (Pbm) less than 1000 µm were examined. The results were standardized for airway size using the Pbm (µm^2^/µm). At least five small airways were counted on each slide. The area of the small airway wall stained with Masson's Trichrome was quantified as described previously [21].

### Immunohistochemistry

Immunohistochemical (IHC) evaluation was performed as described elsewhere [22]. Tissue sections were deparaffinized, rehydrated and blocked with H_2_O_2_ (3%) for 15 minutes, followed by antigen retrieval performed with citrate buffer (10 mM, pH 6.0) for 15 minutes in a microwave. Tissue sections were treated with normal goat serum (5%) at room temperature (RT). Subsequently, the sections were incubated overnight at 4°C with primary antibodies against MMP9, MMP2, type I collagen, FSP1, vimentin, E-cadherin (Abcam; Cambridge, UK), TIMP1 (Millipore; USA), and α-SMA (Sigma-Aldrich). A horseradish peroxidase (HRP)-conjugated secondary antibody was used and visualized with diaminobenzidine using a Immunohistochemistry Detection Kit (Gene Tech; Shanghai, China), according to the manufacturer's protocols. Double IHC staining of E-cadherin (BD Biosciences; California, USA) and vimentin or FSP1 (Abcam; Cambridge, UK) was performed using the MultiVision polymer detection system (Thermo Scientific; Utah, USA) according to the manufacturer's protocols.

Photographs were taken using identical conditions for light setting and contrast. The color segmentation protocols were utilized in the IPP6.0 system to obtain the data. The area of α-SMA and type I collagen immunostaining in the small airway wall was quantitatively analyzed [23]. The expression of MMP9, MMP2 and TIMP1 in the small airway wall was quantified by measuring the integrated optical density (IOD) of the positive staining area. The number of fibroblasts in the small airway wall was quantified as the number of positively stained FSP1 and spindle-shaped cells per µm length of basement membrane.

### Western Blot Analysis

The lung protein levels of MMP9 and MMP2 were evaluated by Western blotting as previously described [24]. After the lung tissues were lysed in RIPA buffer (Pierce Biotechnology; Rockford, USA), the proteins were quantified using a Pierce BCA protein assay kit (Thermo Scientific; Utah, USA). Equal amounts of total protein were resolved on 8% SDS-PAGE electrophoresis. Following electrophoretic transfer, the membranes were treated at RT for 1 hour with 5% skim milk. Then, the membranes were incubated overnight at 4°C with primary antibodies against MMP9, MMP2 (Abcam; Cambridge, UK), and β-tubulin (Santa Cruz Biotechnology; California, USA). The membranes were then incubated with secondary antibodies conjugated to HRP (Santa Cruz Biotechnology; California, USA). Immunodetection was performed by chemiluminescence (ECL, Millipore). Relative protein levels were quantified and normalized by β-tubulin protein levels. Total cellular extracts were obtained and Western blotting was performed as described previously [25].

### MMP-9 and TIMP-1 measurement by ELISA

The levels of serum MMP-9 and TIMP-1 were detected using MMP-9 and TIMP-1 ELISA kits (R&D Systems; Minneapolis, USA). All samples were measured in duplicate, and the average values were used.

### Cell Culture Stimulated by Wood Smoke Condensate and Immunofluorescence

Wood smoke condensate was obtained as previously described [18]. Primary rat tracheal epithelial cells (ATCC; Manassas, VA, US) were cultured in a humidified atmosphere of 5% CO_2_ at 37°C. Prior to the experiments, the cells were serum starved for 24 h and then stimulated with 10 µg/ml of wood smoke condensate diluted in growth medium (containing 0.3% FBS) for 7 days. Then, the cells were harvested for immunofluorescence,cell lysis and protein extraction. The cells were fixed in 4% (w/v) paraformaldehyde for 15 minutes and were then incubated with primary antibodies (E-cadherin, vimentin, Type I collagen) and detected using an appropriate fluorochrome- linked secondary antibody. DAPI was used as a nuclear counterstain. Images were acquired using a Zeiss Axio Imager 2 Microscope (Carl Zeiss, Germany).

### Statistical Analysis

The data were expressed as the mean ± SEM. Statistical analysis was performed with the statistics package SPSS18.0 (SPSS Inc., Chicago, USA). Multiple group comparisons were performed using one-way ANOVA. Statistical significance was set at p<0.05.

## Results

### Histological Morphometric Analysis

**Figure 2:**
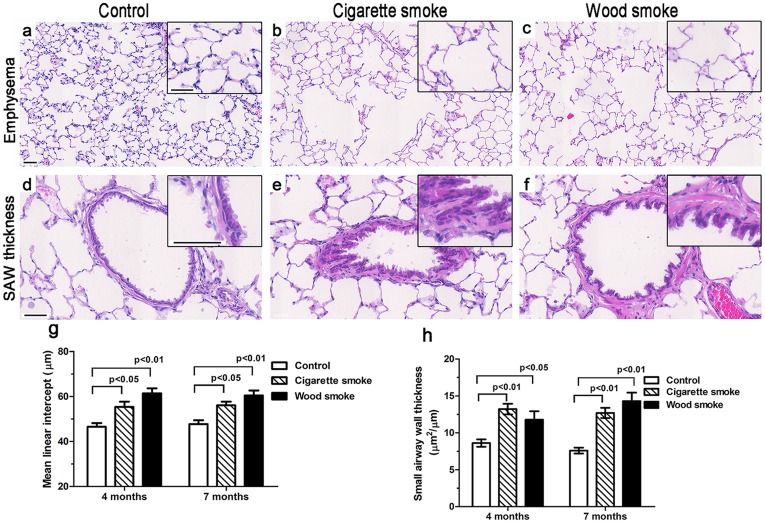
The airspace enlargement and small airway wall thickening in rats exposed to wood smoke. (a–c, g) Photomicrograph and graphical data demonstrating that the airspace size increased significantly in rats exposed to WS or CS for 7 months. (d–f, h) Photomicrograph and graphical data showing that the thickness of the small airway wall (SAW) increased significantly in rats exposed to WS or CS for 7 months. Data are shown as the mean ± SEM. n = 8 animals/group. Overall comparisons were made using one-way ANOVA (p<0.05). Scale bar  = 50 µm.

Histological analysis revealed the presence of emphysematous lesions and small airway wall thickening in rats exposed to smoke (Figures 2a–f). At 7 months, the mean linear intercept difference was significant between the controls (47.7±4.0 µm) and the WS group (60.5±6.7 µm, p<0.01) or the CS group (56.1±4.1 µm, p<0.05) (Figure 2g). The difference in the thicknesses of the small airway wall was markedly significant between the controls (7.6±1.1 µm^2^/µm) and the WS group (14.3±3.3 µm^2^/µm, p<0.01) or the CS group (12.1±3.4 µm^2^/µm, p<0.01) (Figure 2h).

### Airway Smooth Muscle Thickness

**Figure 3:**
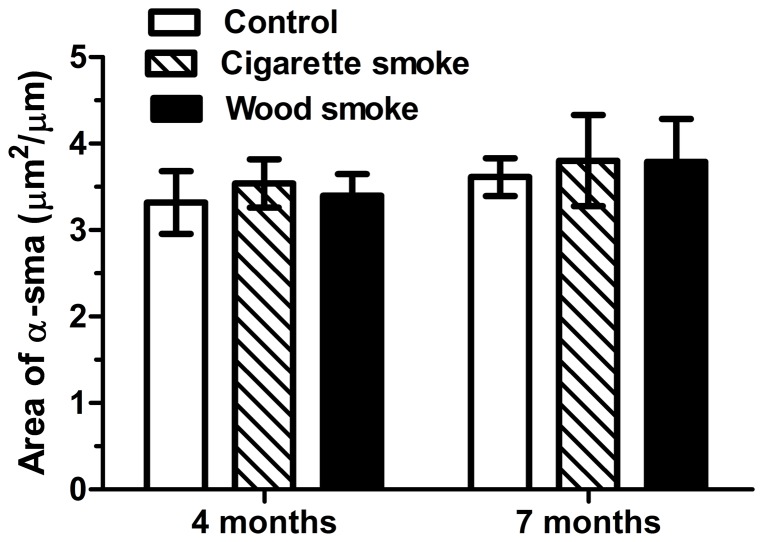
The thickness of airway smooth muscle in small airway wall in rats exposed to wood smoke. The stained area of smooth muscle around the small airway wall was not significantly different among the groups. Data are shown as the mean ± SEM. n = 8 animals/group.

The thickness of the airway smooth muscle was quantified using the area of α-SMA IHC staining. Neither WS nor CS induced a statistically significant increase in the thickness of the small airway smooth muscle (p>0.05) (Figure 3).

### Increased Collagen Deposition in Small Airway Walls

**Figure 4:**
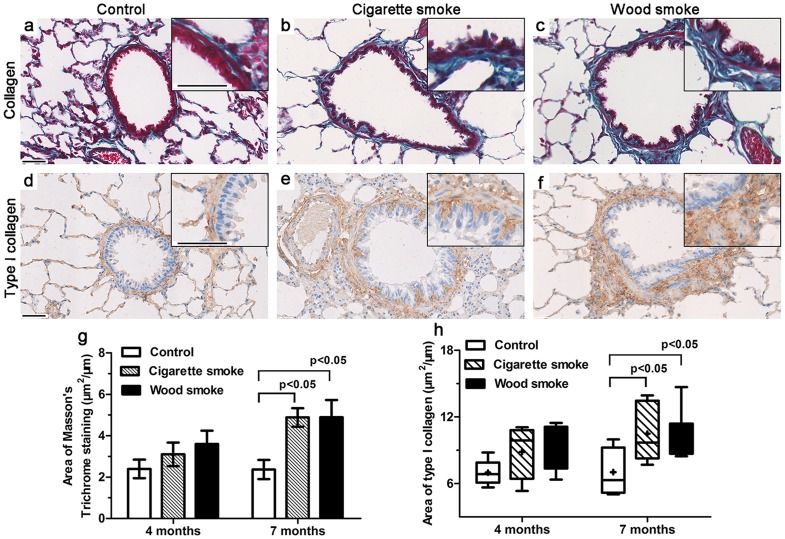
Increased collagen deposition in the small airways was induced by wood smoke. (a–f) Masson's Trichrome staining and IHC staining for Type I collagen showed increased collagen deposition in the small airway wall after 7 months of smoke exposure. (g, h) Graphical data showed that the area of total collagen and type I collagen deposition in the small airway wall exposed to WS or CS was significantly increased compared to controls at 7 months, but there was no significant difference at 4 months. Data are shown as the mean ± SEM or as box and whisker plots with the median, minimum and maximum values. n = 8 animals/group. Scale bar  = 50 µm.

The change in the level of small airway fibrosis was assessed by Masson's Trichrome staining and type-I collagen IHC staining methods (Figures 4a–f). After 4 months of smoke exposure, there were no significant differences in collagen deposition between the groups. At 7 months, both the WS group (4.9±2.2 µm^2^/µm, p<0.05) and the CS group (4.9±1.0 µm^2^/µm, p<0.05) exhibited significantly more collagen deposition in the small airway wall than the controls (2.4±1.1 µm^2^/µm) (Figure 4g). Additionally, the area of type-I collagen staining in the small airway wall was significantly higher in the WS group (11.1±2.7 µm^2^/µm, p<0.05) and the CS group (10.5±2.5 µm^2^/µm, p<0.05) than in controls (7.0±2.1 µm^2^/µm) (Figure 4h).

### Increased Expression of MMP9, MMP2 and TIMP1

**Figure 5:**
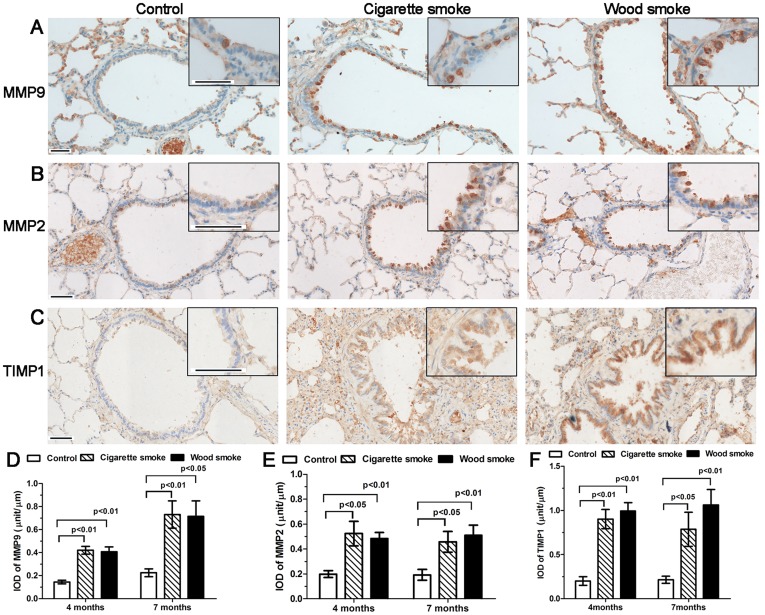
Increased immunostaining of MMP9, MMP2 and TIMP1 in the small airway wall secondary to wood smoke exposure. (A–C) Photomicrograph data showing the IHC staining pattern for MMP9, MMP2 and TIMP1 in small airway walls exposed to smoke for 7 months. MMP9, MMP2 and TIMP1 immunostaining was primarily localized in airway epithelial cells, infiltrating inflammatory cells and fibroblasts. (g, h) Graph showing that the IODs of MMP9, MMP2 and TIMP1 increased significantly in the small airway wall secondary to WS or CS exposure for 4 to 7 months. Data are shown as the mean ± SEM. n = 8 animals/group. Scale bar  = 50 µm.

MMP9, MMP2 and TIMP1 immunostaining was primarily localized in airway epithelial cells, as well as infiltrating inflammatory cells and fibroblasts (Figures 5A–C). Moreover, the IODs of MMP9, MMP2 and TIMP1 immunostaining in the WS group and the CS group were significantly higher than those in controls at 4 or 7 months (Figures 5D–F).

**Figure 6:**
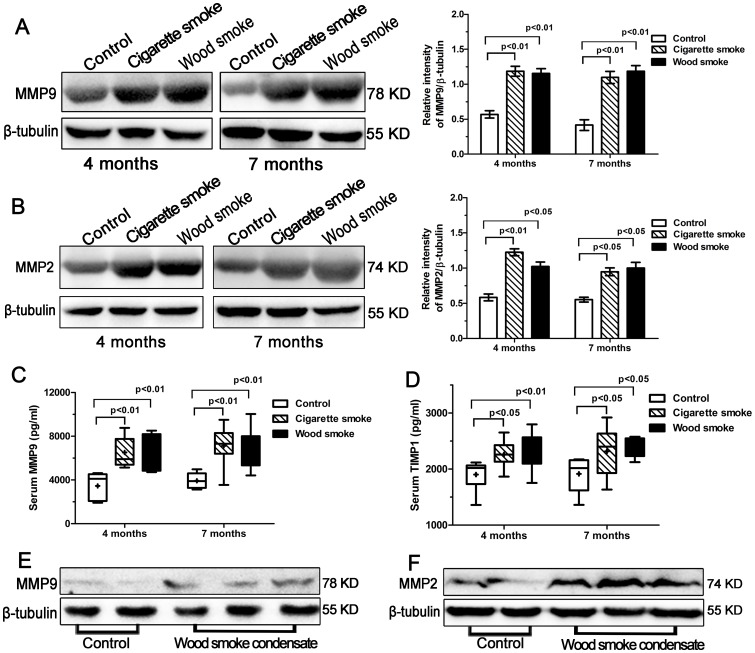
The increased expression of MMP9, MMP2 and TIMP1 by Western blotting or ELISA. Western blot analysis of MMP9 protein expression (78 KD) in the lung tissues from each group is shown in (A); MMP2 protein expression (74 KD) in (B). Graph showed that the relative intensity of MMP9/β-tubulin protein expression or MMP2/β-tubulin protein expression was higher in lung tissues after smoke exposure for 4 to 7 months compared to controls. (C, D) The levels of serum MMP9 and TIMP1 were significantly higher in the WS group and the CS group than in controls. (E, F) Wood smoke condensate induced an increased expression of MMP9 and MMP2 proteins in primary rat tracheal epithelial cells by Western blotting. Data are shown as the mean ± SEM or as box and whisker plots with the median, minimum and maximum values. n = 8 animals/group.

In whole lung tissue and cellular protein extraction, western blotting revealed lysis bands of estimated molecular weights of 78 KD (MMP9), 74 KD (MMP2) and 55 KD (β-tubulin) in the samples. The expression levels of MMP9 and MMP2 in the WS group and the CS group were markedly increased in comparison with controls (Figures 6A and 6B). And wood smoke condensate induced the increased expression of MMP9 and MMP2 proteins in primary rat tracheal epithelial cells (Figures 6E and 6F).

### The Serum Levels of MMP9 and TIMP1

At 7 months, the levels of serum MMP9 and TIMP1 were significantly higher in the WS group and the CS group than in controls, but there were no significant differences between the WS group and the CS group (Figures 6C and 6D).

### Increased Fibroblasts in Small Airway Walls

**Figure 7:**
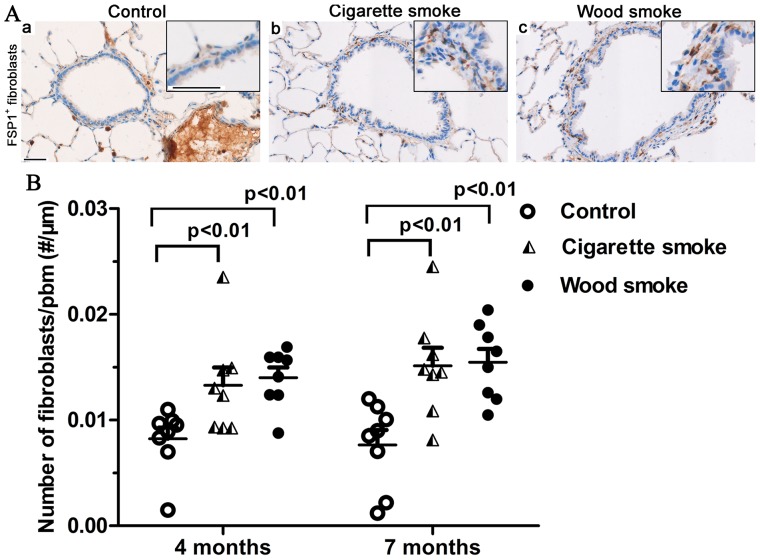
Increased fibroblasts in small airway walls in rats induced by wood smoke exposure. Immunostaining for FSP1 and spindle-shaped cells in small airway walls following smoke exposure for 7 months is shown in (A). (B) Graph showing that the number of fibroblasts increased significantly in small airway walls exposed to WS or CS for 4 to 7 months. Data are shown as the mean ± SEM. n = 8 animals/group. Scale bar  = 50 µm.

Immunostaining for anti-FSP1 clearly demonstrated that spindle-shaped fibroblasts were present in the small airway subepithelium and adventitial compartment (Figure 7A). The number of fibroblasts in the small airway wall was significantly higher in the WS group and the CS group than in controls (Figure 7B).

### Expression of EMT Markers

**Figure 8:**
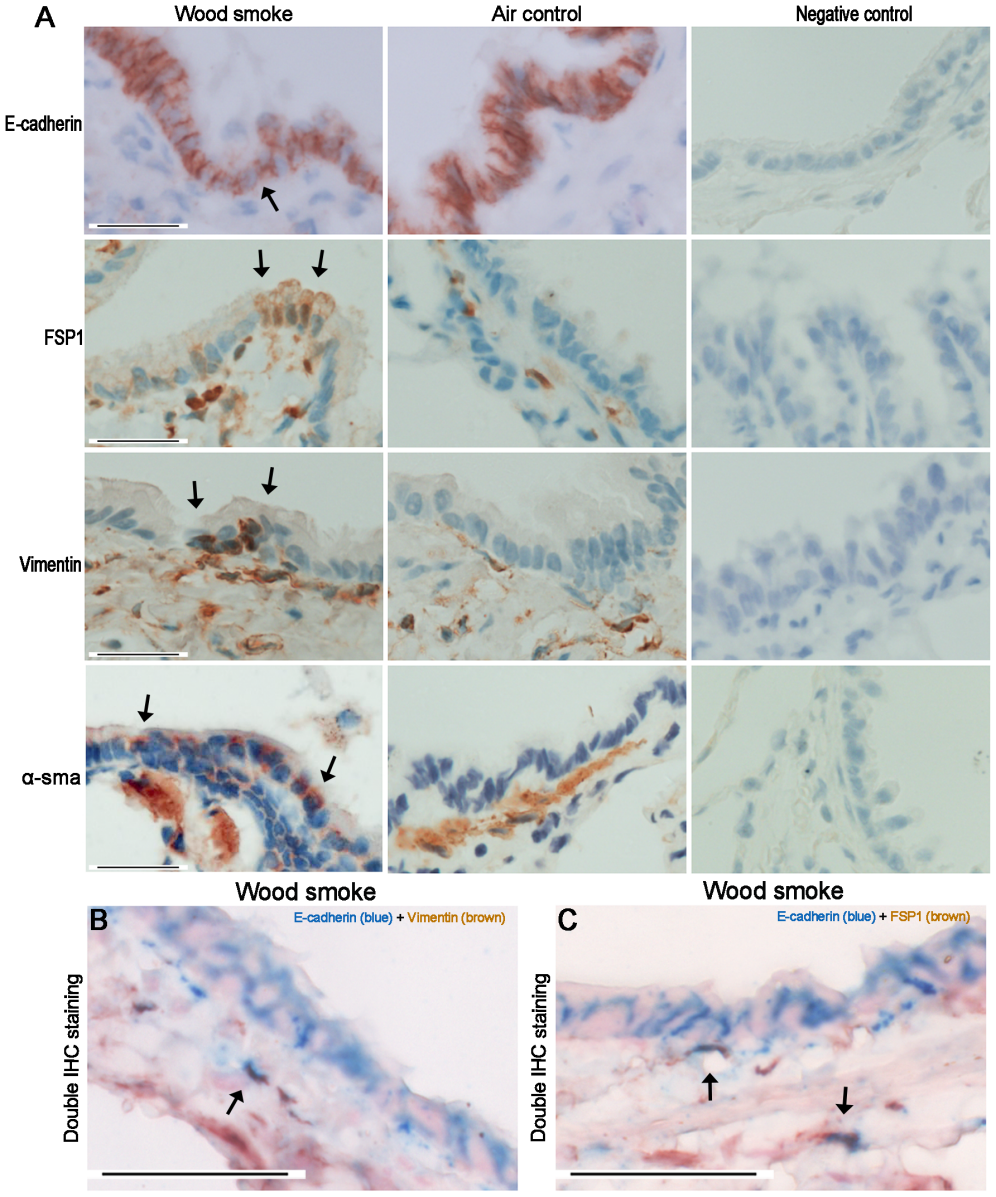
Expression of EMT Markers in Small Airway Walls Exposed to wood smoke. (A) Photomicrographs showing that the small airways immunostained for E-cadherin, FSP1, vimentin and α-SMA. Although the airway epithelium in rats exposed to WS did not show a marked decrease in E-cadherin immunostaining compared to controls, the positive staining of mesenchymal markers (FSP1, vimentin, α-SMA) was sometimes observed in the small airway epithelium at 7 months. (B, C) Photomicrographs showing the small number of cells that double immunostained for E-cadherin and vimentin or FSP1 in the airway subepithelium of rats exposed to WS for 7 months (only 3 of these 8 rats). Black arrows show positively immunostained cells. Scale bar  = 50 µm.

**Figure 9:**
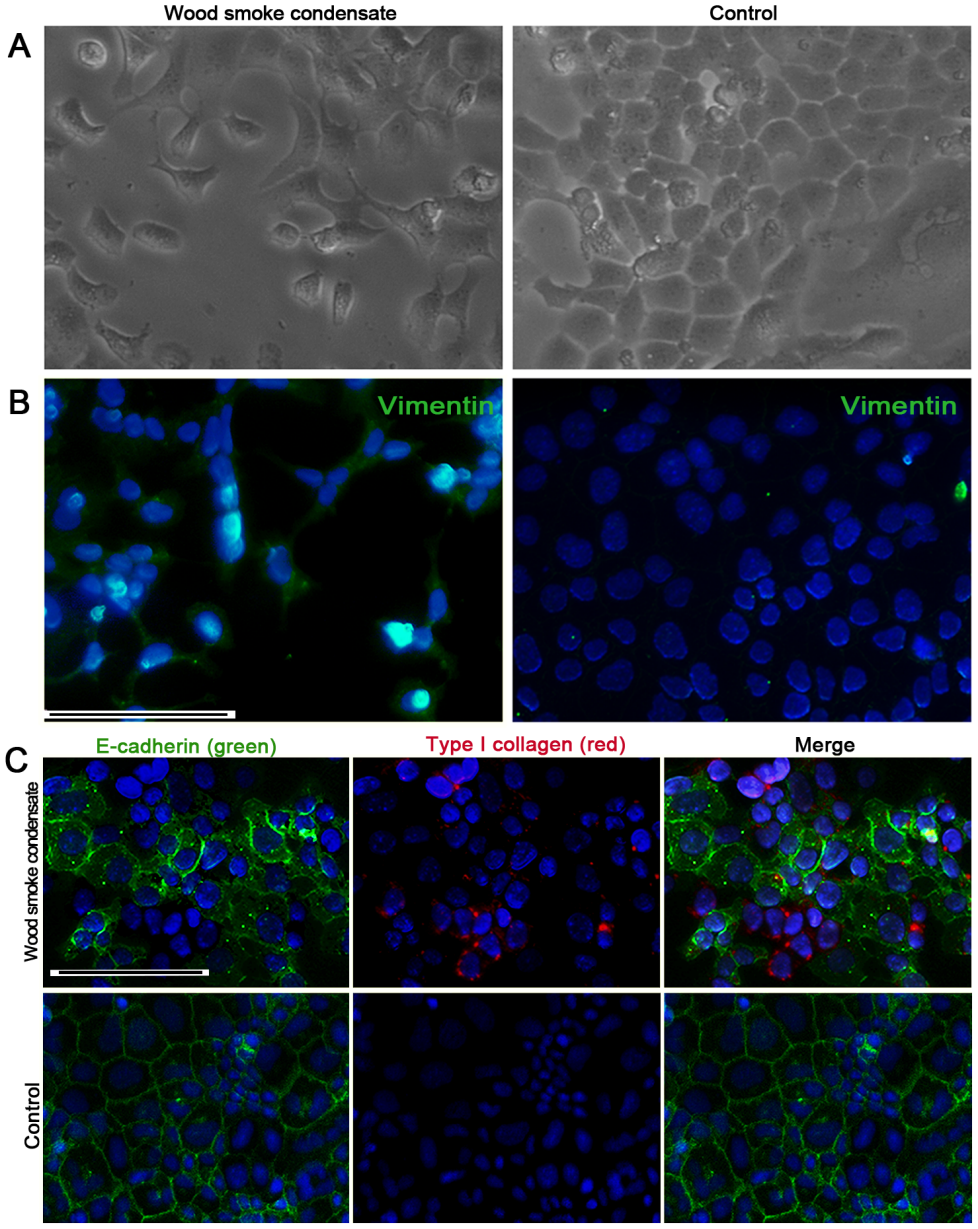
Wood smoke condensate induced EMT-related changes in primary rat tracheal epithelial cells. (A) Wood smoke condensate induced a mesenchymal morphology. (B, C) Immunofluorescent staining showed that wood smoke condensate induced a downregulation of the epithelial marker (E-cadherin) and an upregulation of the mesenchymal markers (vimentin and type I collagen) in primary rat tracheal epithelial cells. Scale bar  = 50 µm.

Although the airway epithelium in rats following exposure to WS did not show a marked decrease in E-cadherin immunostaining compared with controls, positive staining for mesenchymal markers (FSP1, vimentin, α-SMA) was sometimes observed in the small airway epithelium at 7 months (Figure 8A). Furthermore, Figures 8B and 8C showed the small number of cells that double immunostained for E-cadherin and vimentin or FSP1 in the airway subepithelium of rats exposed to WS at 7 months (only 3 of these 8 rats). In vitro, primary rat tracheal epithelial cells had a cobble stone morphology, but a morphological phenotype characteristic of EMT was observed in the presence of wood smoke condensate, with loss of cell-cell contact and an elongated shape (Figure 9A). In addition, immunofluorescent staining showed that the expression of E-cadherin decreased in primary rat tracheal epithelial cells after 7 days of treatment with wood smoke condensate, and the expression of vimentin and type I collagen was observed (Figures 9B and 9C).

## Discussion

The household use of wood and other biomass fuels is a frequent practice worldwide, but the respiratory effects of chronic exposure to WS have scarcely been investigated [26]. Although SAR is an important cause of airflow obstruction, very little is known about its pathogenesis. The present study showed that the pathological changes in the rat respiratory tract following chronic exposure to WS were similar to those in rats exposed to CS. The expression of MMP9, MMP2 and TIMP1 was markedly increased in the small airway walls of rats exposed to WS, and the expression was primarily located in the airway epithelium. Moreover, the serum MMP9 and TIMP1 concentrations were significantly increased in rats exposed to WS or CS. The number of fibroblasts was markedly increased in the subepithelium and outer walls of small airways chronically exposed to WS. Staining for mesenchymal markers and double IHC staining showed that partial airway fibroblasts may derive directly from bronchial epithelial cells via EMT. In vitro, the expression of MMP9 and MMP2 proteins was upregulated in primary rat tracheal epithelial cells following exposure to wood smoke condensate for 7 days; and positive immunofluorescent staining for vimentin and type I collagen was also observed. Our results suggest that gelatinases and EMT might play a role in SAR in COPD induced by chronic WS exposure.

China firs are widely used for daily cooking in the rural areas of China. CO is an indicator of wood smoke exposure and the largest constituent of incomplete combustion [27]. A baseline survey in Zimbabwe showed that the levels of CO in the kitchen during cooking were in the range of 300–1,000 ppm [28]. In our study, the CO concentration in rats exposed to WS was consistent with those levels.

The present study demonstrated that the COPD-like pathological alterations in rats exposed to WS were similar to those in rats exposed to CS, consistent with the results of a previous human study [29]. Wood smoke contains thousands of chemical components, including fine particles, CO, nitrogen oxides, respiratory irritants and other toxic organic compounds, which have adverse effects on human health [30]. Previous research has shown that women exposed to biomass smoke can develop airflow limitation [31]. SAR leads to airway wall thickening and fixed airway obstruction [5]. In the present study, although there was a small increase in smooth muscle thickness in rats exposed to smoke, the difference was not significant. In contrast to asthma, the airway smooth muscle in COPD patients may play a relatively minor role in airway remodeling. Our results demonstrated that the amounts of both total collagen and type I collagen were significantly increase in small airways chronically exposed to WS. Type I collagen is a major matrix element that resists tensile stresses in the lung, and thus, alterations in type I collagen at the distal level might contribute to airway-parenchyma structural changes, such as airway obstruction [32].

The present study showed that the expression of MMP9, MMP2 and TIMP1 was markedly increased in small airway walls and whole lung tissue in rats exposed to smoke; serum MMP9 and TIMP1 concentrations were also significantly higher in rats secondary to WS or CS when compared with controls. In vitro, wood smoke condensate induced the increased expression of MMP9 and MMP2 proteins in primary rat tracheal epithelial cells. Montaño et al. had demonstrated that the expression and gelatinolytic activity of MMP9 and MMP2 were significantly increased in bronchoalveolar lavage fluid from COPD patient associated with wood smoke [26]. Carlos Ramos et al. [18] reported that increases in MMP9 and MMP2 activity and expression could be responsible for emphysematous lesions induced by wood smoke exposure in guinea pigs. The present results are in accordance with those conclusions, which the upregulation of MMP9 and MMP2 could lead to pulmonary emphysema in rats exposed to WS. Furthermore, our results suggest that the increased expression of MMP9 and MMP2 might play a role in collagen deposition in small airway walls in rats. This might seem counterintuitive since proteinases degrade matrix. However, the net effect on collagen depends on whether a particular proteinase can degrade collagen as well as other non-matrix substrates [33]. It has been reported that MMP9 and to a lesser extent MMP2, can cleave a variety of non-ECM proteins, such as certain chemokines, growth factors and amyloid beta peptide [34]. In addition, MMP9 and MMP2 can directly process TGF-β into an active ligand and induce TGF-β1 production in the airway epithelium and peribronchiolar fibroblast proliferation [35]. TIMP1 is significantly higher in rats exposed to wood smoke than in control subjects, suggesting the existence of a protease–antiprotease imbalance. Deregulation of matrix metalloproteinases activities is a dynamic course, and leads to states of impaired repair and pathological tissue remodeling [36]. Our results demonstrated that MMP9, MMP2 were mainly synthesized and secreted by airway epithelial cells. This study provided evidence indicating that the airway epithelium is a direct and main source of gelatinases in the small airway wall following to WS exposure. Therefore, MMP inhibitors administered by aerosol delivery might be an effective and safe treatment method, permitting local administration and a lower dose of medicine [37]. The use of specific MMP inhibitors or knock-out mice will be of interest in the future to assess whether MMP9/MMP2 are crucial in wood-smoke induced development of COPD manifestations.

Fibroblasts and epithelial cells are the main effector cells during airway remodeling [38]. A significant increase in the number of fibroblasts is observed in small airway walls following exposure to WS or CS. It has been established that fibroblast accumulation may occur as a consequence of resident fibroblast to myofibroblast transitions, the recruitment of circulating fibroblastic stem cells or EMT [39]. FSP1, vimentin and α-SMA are widely used as mesenchymal biomarkers of EMT. In rats exposed to WS for 7 months, the positive staining of mesenchymal markers was sometimes observed in the airway epithelium; few cells that double immunostained for E-cadherin (epithelial marker) and vimentin or FSP1 were observed in the airway subepithelium. In vitro, primary rat tracheal epithelial cells stimulated by wood smoke condensate presented a morphological phenotype characteristic of EMT; immunofluorescent staining showed that the expression of E-cadherin had decreased, and the expression of vimentin and type I collagen was observed. These results indicate that partial bronchial fibroblasts might originate directly from epithelial cells via EMT. A previous study in patients with cigarette smoke-induced COPD has shown that E-cadherin expression in the airway epithelium is significantly decreased during EMT [17]. However, our results illustrate that E-cadherin immunostaining in rats exposed to WS was not markedly down-regulated; double positive IHC staining cells were found in only 3 of these 8 rats. We speculate that the wood smoke exposure index was not sufficiently strong; moreover, EMT is a long-term and dynamic process. It has been reported that the reticular basement membranes of smokers and COPD patients are highly fragmented with elongated spaces or cracks [40]. MMP9 and MMP2 contain fibronectin type II-like repeats within their catalytic domains and primarily degrade type IV collagens, which are thought to assist epithelial cell migration by disrupting the underlying basement membrane [41]. In addition, the present study demonstrated the destruction of alveolar structure, but mild fibrosis in lung tissues. We did not performed the double immunostaining with surfactant protein C (a biomarker of alveolar epithelial cell) and mesenchymal biomarkers. Unlike normal human alveolar type II epithelial cell, those isolated from the lungs of patients with idopathic pulmonary fibrosis express higher levels of mRNA for the mesenchymal proteins, which suggests the human alveolar type II epithelial cells could acquire features of mesenchymal cells via EMT. However, there are no clinical and experimental researches on the relationship between EMT and emphysema yet.

In summary, chronic exposure to WS produces COPD-like pathological alterations similar to CS in a rat model. The increased expression of MMP9 and MMP2 in small airway walls exposed to WS appears to play a role in peribronchiolar fibrosis, which then leads to small airway wall thickening. EMT is also likely involved in wood smoke-induced SAR. Understanding the roles that gelatinases and EMT play in SAR may aid the treatment of patients with COPD associated with chronic WS exposure.
